# Extended venous thromboembolism prophylaxis after bariatric surgery: the potential role of aspirin

**DOI:** 10.1097/MS9.0000000000005173

**Published:** 2026-05-20

**Authors:** Michele Carron

**Affiliations:** Department of Medicine – DIMED, Section of Anesthesiology and Intensive Care, University of Padua, Padua, Italy

**Keywords:** anticoagulation, aspirin, bariatric surgery, prophylaxis, venous thromboembolism

## Abstract

Extended venous thromboembolism (VTE) prophylaxis after bariatric surgery remains an unresolved clinical issue. Current guidelines acknowledge the persistence of thrombotic risk beyond hospital discharge but do not define a standardized duration of pharmacological prophylaxis. In this perspective, a sequential strategy consisting of perioperative low-molecular-weight heparin followed by post-discharge aspirin is presented as a biologically plausible and pragmatically testable strategy for extended VTE prophylaxis in selected higher-risk bariatric patients. Persistent postoperative platelet hyperreactivity may contribute to sustained thrombotic risk after hospital discharge, providing a mechanistic basis for antiplatelet therapy during the extended prophylaxis phase. Although evidence supporting aspirin in this setting remains indirect, its oral administration, potential effects on platelet-mediated thrombosis, and favorable adherence profile support its evaluation as a hypothesis-generating strategy in this population.

Patients with obesity present a chronic pro-thrombotic state, driven by inflammation, endothelial dysfunction, hypercoagulability, impaired fibrinolysis, and increased platelet reactivity^[^1^]^. Consequently, venous thromboembolism (VTE) remains one of the most feared complications after bariatric surgery, contributing to morbidity and mortality despite advances in perioperative care, and effective VTE prophylaxis remains essential^[^2–4^]^. However, the optimal duration and strategy of prophylaxis remain unresolved and clinically relevant issues^[^2–4^]^, with substantial variability across guidelines and clinical practice^[^2–5^]^. In this context, a sequential strategy consisting of perioperative low-molecular-weight heparin (LMWH) followed by post-discharge aspirin may represent a biologically plausible and pragmatically testable strategy for extended VTE prophylaxis in selected higher-risk bariatric patients and is presented here as a hypothesis-generating clinical perspective rather than a prescriptive strategy. International guidelines consistently recommend pharmacological thromboprophylaxis for bariatric surgery, typically based on LMWH, with or without mechanical methods^[^2–4^]^. However, they diverge regarding duration and specific regimens. The European Society of Anesthesiology suggests extending prophylaxis for 10–15 days in high-risk patients^[^3^]^, while the 2019 American Society of Hematology guidelines distinguish between short-term (4–14 days) and extended prophylaxis (>3 weeks, 19–42 days), recommending prolonged strategies in patients with additional risk factors^[^4^]^. Despite this, no standardized approach is defined, and decisions are largely left to individual risk stratification^[^2–4^]^. This uncertainty is reflected in clinical practice, with substantial variability in prophylaxis duration. A recent survey showed striking variability, with 38.7% of surgeons prescribing LMWH for 2 weeks, 28.9% for 4 weeks, and others limiting prophylaxis to hospitalization, underscoring the absence of a unified standard^[^5^]^. Moreover, large registry analyses have identified multiple risk factors – such as prior VTE, oxygen dependence, immobility, higher BMI, longer operative time, dialysis, chronic steroid use, venous stasis, and vena cava filter – associated with postoperative pulmonary embolism^[^6^]^, largely overlapping with those recognized in guideline recommendations (advanced age, high BMI, prior VTE, open or revisional surgery, reduced mobility, and obstructive sleep apnea syndrome)^[^2–4^]^. Taken together, these factors delineate a broad spectrum of risk conditions that support the rationale for extended or intensified prophylaxis^[^2–4,6^]^. In addition to these risk factors, the temporal distribution of events underscores that thrombotic risk after bariatric surgery extends well beyond hospitalization. Froehling *et al* reported a cumulative incidence of VTE after bariatric surgery of 0.3% at 7 days, 1.9% at 30 days, and 2.1% at both 3 and 6 months, with nearly 80% of events occurring after discharge^[^7^]^. Notably, this temporal distribution contrasts with other surgical populations, where thrombotic risk typically peaks in the immediate postoperative period and subsequently declines^[^8^]^.

Pathophysiological data reinforce this concern: thromboelastography showed that, despite LMWH preserving coagulation and fibrinolysis, patients developed progressively increased platelet activity postoperatively^[^9^]^. This persistent platelet hyper-reactivity may contribute to ongoing thrombotic risk, suggesting that an antiplatelet strategy could complement or sequentially follow LMWH^[^9^]^. Supporting this biological rationale, a pilot study evaluating aspirin pharmacodynamics before and after bariatric surgery demonstrated enhanced aspirin-induced platelet inhibition following surgery, suggesting that weight-loss-related metabolic changes may enhance platelet responsiveness to antiplatelet therapy^[^10^]^. In this context, a sequential strategy combining perioperative LMWH with a subsequent antiplatelet approach may be considered as a hypothesis-generating strategy for extended prophylaxis in selected bariatric patients^[^11^]^.

Aspirin is an inexpensive, oral antithrombotic agent that has been evaluated for extended VTE prevention. A recent meta-analysis confirmed that aspirin (100–160 mg) significantly reduces VTE, including deep vein thrombosis and pulmonary embolism, as well as VTE-related mortality in primary prevention and provoked settings^[^11^]^. The translation of evidence from orthopedic or other non-bariatric surgical populations – where thrombotic risk is largely driven by local factors such as tissue injury and immobilization – to the bariatric setting requires careful contextualization rather than direct extrapolation, as this population is characterized by a persistent prothrombotic milieu driven by inflammation, endothelial dysfunction, and enhanced platelet reactivity, together with postoperative metabolic and anatomical changes that may influence drug absorption and pharmacodynamic response^[^1^]^. Within this pathophysiological framework, antiplatelet strategies may be particularly relevant, although their role in this specific setting remains to be clearly defined. No randomized controlled trials have specifically evaluated a sequential strategy of perioperative LMWH followed by aspirin in bariatric surgery^[^11^]^. Therefore, the applicability of existing aspirin evidence to bariatric surgery should be considered indirect and hypothesis generating rather than practice defining. From a pragmatic perspective, this strategy might be most reasonably explored in bariatric patients with a higher baseline thrombotic burden or multiple cumulative risk factors, including those previously identified in guideline recommendations and registry analyses – such as prior VTE, elevated BMI (particularly BMI >50 kg/m^2^), revisional or open bariatric procedures, reduced mobility, and other clinical factors associated with postoperative thromboembolic risk – although no validated risk threshold currently exists^[^2–4,6^]^. To enhance clinical interpretability, a pragmatic, risk-oriented framework may be considered to contextualize patient selection. Bariatric patients may be conceptually stratified into three broad categories based on cumulative thrombotic risk:

- Low-risk profile: BMI <40 kg/m^2^, no major risk factors, and early postoperative mobilization.
- Intermediate-risk profile: BMI 40–50 kg/m^2^, particularly in the presence of additional risk factors (e.g., limited mobility or relevant comorbidities).
- High-risk profile: BMI >50 kg/m^2^, revisional or open bariatric surgery, prior VTE, immobility, or multiple cumulative risk factors^[^2–4,6^]^.

Within this conceptual framework, a sequential LMWH-to-aspirin strategy may be more reasonably explored in intermediate- to high-risk patients, while low-risk profiles may be adequately managed with standard prophylactic approaches^[^2–4,6^]^. Extended prophylaxis after bariatric surgery may rely on continued LMWH, oral anticoagulants such as direct oral anticoagulants (DOACs), or a sequential antiplatelet strategy^[^12,13^]^. LMWH remains the most established approach but may be limited by prolonged injections and adherence issues in the post-discharge setting^[^11^]^. DOACs offer oral administration, but their use after bariatric surgery raises concerns regarding altered absorption and pharmacokinetics due to surgical modifications of the gastrointestinal tract, including changes in gastric pH, intestinal transit time, and the absorptive surface of the proximal intestine^[^12^]^. These pharmacokinetic considerations in patients with obesity have also been discussed in a recent European Society of Cardiology clinical consensus statement on antithrombotic therapy and body mass, which highlights the limited evidence available and the need for careful contextualization when applying anticoagulant strategies in this population^[^14^]^. Consistent with this uncertainty, emerging evidence suggests that DOACs may represent a potentially feasible strategy for thromboprophylaxis after bariatric surgery, although further studies are required to establish definitive recommendations^[^13^]^. In this context, a sequential LMWH-to-aspirin strategy may represent a pragmatic alternative for extended prophylaxis, particularly when prolonged anticoagulation is impractical or poorly tolerated^[^11^]^. Overall, these strategies offer complementary profiles: LMWH provides established efficacy but limited adherence, DOACs ensure oral administration with uncertain pharmacokinetic reliability, while aspirin represents a simple option supported by indirect evidence and requiring careful patient selection (Fig. 1).

**Figure 1.**
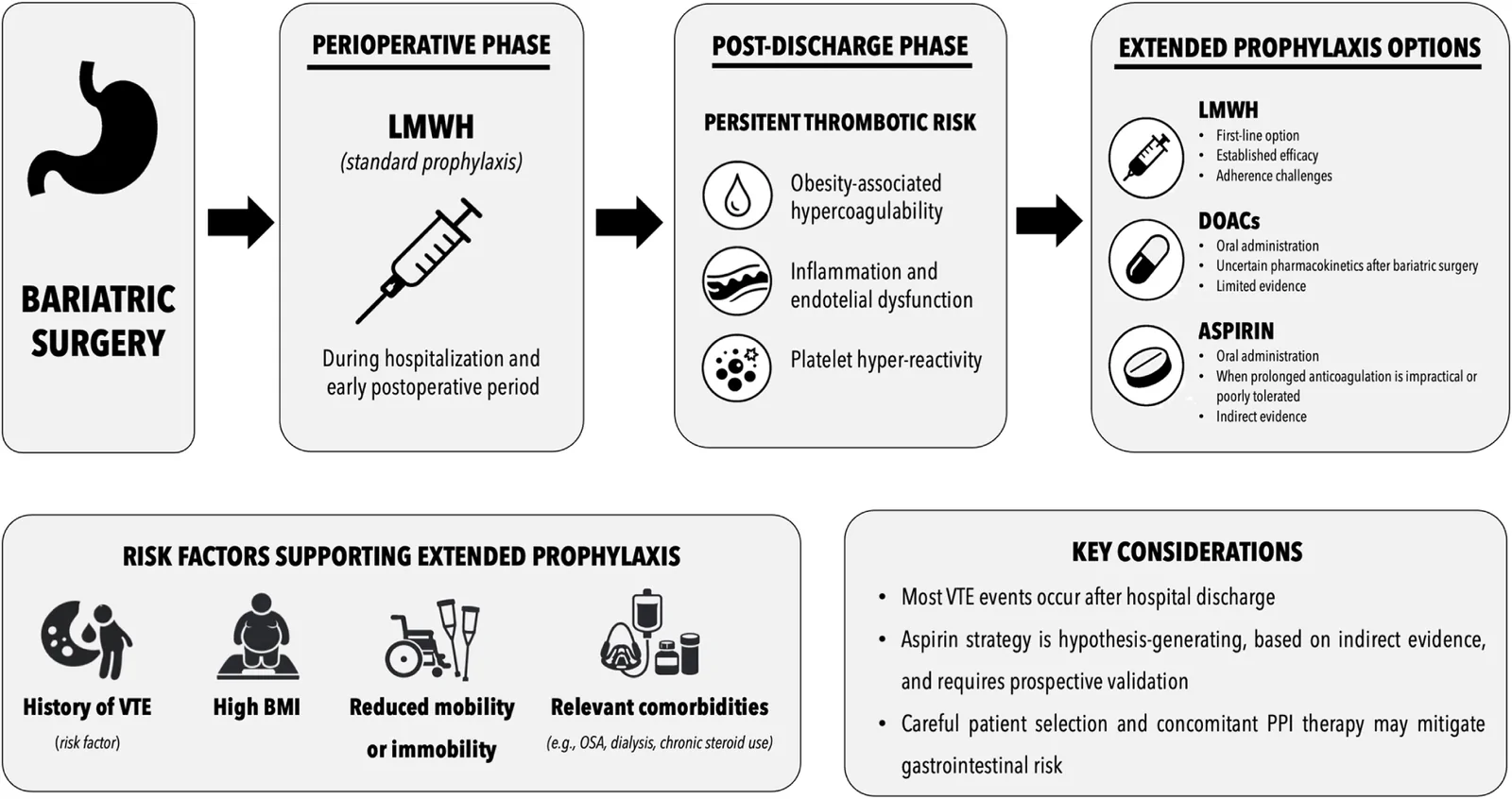
Conceptual framework of sequential thromboprophylaxis after bariatric surgery. This schematic illustrates a conceptual, hypothesis-generating framework for extended thromboprophylaxis after bariatric surgery. The sequential use of aspirin following perioperative LMWH is presented as a potential strategy in selected higher-risk patients and should not be interpreted as a validated clinical algorithm. BMI, body mass index; DOACs, direct oral anticoagulants; LMWH, low-molecular-weight heparin; OSA, obstructive sleep apnea; PPI, proton pump inhibitor; VTE, venous thromboembolism.

Although aspirin may increase the risk of bleeding, available evidence suggests that this does not translate into a clinically meaningful excess of major bleeding events^[^11^]^. In addition, number-needed-to-treat versus number-needed-to-harm analyses support a favorable benefit–risk profile of extended aspirin, particularly in higher-risk and provoked settings^[^11^]^. However, the potential gastrointestinal vulnerability associated with bariatric procedures should be considered, particularly in the presence of staple lines or anastomoses (such as the gastrojejunal anastomosis after Roux-en-Y gastric bypass) or altered postoperative anatomy^[^15^]^. Procedure-specific risk appears particularly relevant after Roux-en-Y gastric bypass, where marginal ulceration represents a recognized complication in a susceptible postoperative environment. The pathophysiology is multifactorial and associated with several risk factors, including *Helicobacter pylori* infection, smoking, and diabetes mellitus, while the contribution of NSAID exposure remains inconsistently reported across studies^[^15^]^. Observational evidence suggests that low-dose aspirin use after Roux-en-Y gastric bypass does not appear to significantly increase the incidence of marginal ulceration, although data remain limited^[^16^]^. In this context, careful patient selection – particularly avoiding aspirin in individuals with active ulcer disease or multiple gastrointestinal risk factors – and concomitant proton pump inhibitor therapy may help mitigate gastrointestinal risk when antiplatelet therapy is clinically considered^[^17^]^.

Thus, while individualized risk–benefit assessment remains essential, aspirin may represent a potential sequential option following perioperative LMWH for extended prophylaxis in selected bariatric patients, particularly to address the persistent platelet-driven thrombotic tendency associated with obesity after surgery^[^11^]^. At present, anticoagulant-based extended prophylaxis remains the standard approach when clinically feasible. However, when prolonged anticoagulation is impractical or poorly tolerated, a sequential LMWH-to-aspirin strategy may represent a biologically plausible hypothesis deserving prospective evaluation^[^11^]^. Supporting this rationale, large pragmatic randomized trials conducted in orthopedic trauma populations have shown that thromboprophylaxis with aspirin was noninferior to LMWH for preventing fatal outcomes and was associated with similarly low incidences of pulmonary embolism and overall mortality^[^18^]^. Although these findings arise from a different surgical population, they suggest that antiplatelet strategies may represent a feasible alternative in settings where prolonged anticoagulation is challenging^[^18^]^. Accordingly, future research should focus on pragmatic multicenter randomized trials comparing standard LMWH-based prophylaxis with a sequential LMWH-to-aspirin strategy in intermediate- to high-risk bariatric patients. The target population should include individuals with elevated thrombotic risk (e.g., prior VTE, elevated BMI, comorbidities, major surgery, or limited mobility). Primary efficacy endpoints should assess symptomatic VTE during extended follow-up (30–90 days), while safety endpoints should include major bleeding and reoperation, with secondary outcomes including treatment adherence and cost-effectiveness.

No AI-based tools were used in the preparation of this manuscript, in accordance with the TITAN Guidelines 2025^[^19^]^.

## Data Availability

No new data were generated or analyzed for this article.

## Research registration unique identifying number (UIN)

This study did not involve human participants and, therefore, was not eligible for registration in a clinical or systematic review registry.
